# Green synthesis of silver nanoparticles from plant *Astragalus fasciculifolius* Bioss and evaluating cytotoxic effects on MCF7 human breast cancer cells

**DOI:** 10.1038/s41598-025-05224-5

**Published:** 2025-07-15

**Authors:** Fatemeh Nosrati, Baratali Fakheri, Habib Ghaznavi, Nafiseh Mahdinezhad, Roghayeh Sheervalilou, Bahman Fazeli-Nasab

**Affiliations:** 1 Department of Plant Breeding and Biotechnology, Faculty of Agriculture, University of Zabol, Zabol, Iran; 2 Pharmacology Research Center, Zahedan University of Medical Sciences, Zahedan, Iran; 3 Department of Agronomy and Plant Breeding, Agriculture Institute, Research Institute of Zabol, Zabol, Iran

**Keywords:** Silver nanoparticles (AgNPs), Anzaroot-silver nanoparticles (Anz@AgNPs), Breast cancer, Cytotoxicity, *Astragalus fasciculifolius* (Anzaroot), Biotechnology, Cancer

## Abstract

*Astragalus fasciculifolius* Bioss, commonly known as Anzaroot, is a medicinal plant from the Fabaceae family, recognized for its therapeutic properties due to its rich composition of saponins, flavonoids, and polysaccharides. These compounds have been shown to effectively treat heart diseases and inhibit cancer cell growth while also alleviating chemotherapy side effects. Recent research has focused on the green synthesis of silver nanoparticles (AgNPs) from Anzaroot, exploring their potential cytotoxic effects against MCF-7 human breast cancer cells. This study aims to bridge traditional herbal medicine and modern nanotechnology by evaluating the anticancer properties of AgNPs derived from this lesser-explored plant. This study was aimed to assess the cytotoxic effect of Anzaroot and green synthesized AgNPs using aqueous extract of Anzaroot on the MCF-7 cell line. To optimization of AgNPs synthesis, different parameters were evaluated including Anzaroot aqueous extract volumes (1, 2, 3 and 4 ml), silver nitrate solution (AgNO_3_) concentrations (1, 5 and 10 mM), reaction time (30, 60 and 300 min) and reaction solution pH (2, 4, 6, 8 and 10) at room temperature. Transmission electron microscopy (TEM), Fourier transform infrared (FTIR) spectroscopy, ultraviolet–visible (UV–Vis) spectroscopy, and X-ray diffraction analysis (XRD) were used to characterization of the AgNPs using aqueous extract of Anzaroot-silver nanoparticles (Anz@AgNPs). The obtained Anz@AgNPs exhibited Surface Plasmon Resonance (SPR) centered at 443 nm, with an average particle size calculated to be about 16 nm. The XRD spectrum of Anz@AgNPs showed a face-centred cubic (FCC) crystalline nature. The optimized parameters for successfully AgNPs synthesis were optained as follow: 4 ml aqueous extract volume, 1 and 5 mM AgNO_3_, reaction time of 300 min, and pH 8. MTT assay demonstrated the remarkable dependent dosage anticancer effect of the Anz@AgNPs against MCF-7 cell line. The IC50 value exposed that The lowest and the highest IC50 values was demonstrated for the Anz@AgNPs synthesized through the root extract (21.73 μg/Ml) and the aqueous root extract (348.21 μg/Ml) treatment. Based on the MTT assay, the Anz@AgNPs showed the inhibition of cell proliferation potential more than the aqueous extract of this plant. The plant organs used in Anz@AgNPs synthesis, root and gum, influenced their anticancer activity; nanoparticles synthesized from root extract demonstrated a stronger growth inhibitory effect than those from gum extract. The results demonstrated that anzroot plant can be effectively used as a reducing agent for AgNPs synthesis, and AgNPs have the potential to be used effectively in cancer therapy methods and to inhibit the growth of cancer cells.

## Introduction

The rising prevalence of antibiotic resistance has prompted the need to explore alternative therapeutic strategies, especially in cancer treatment. One promising approach is the green synthesis of silver nanoparticles (AgNPs) using plant extracts, which provides an environmentally friendly and cost-effective solution. *Astragalus fasciculifolius* Bioss (Anzaroot), a plant recognized for its rich phytochemical profile, has demonstrated potential for nanoparticle biosynthesis^1,2^. Antibiotic resistance and cancer treatment are interconnected in several ways, particularly due to the impact of antibiotics on the microbiome and immune system, which can influence cancer therapy outcomes. The gut microbiome plays a crucial role in modulating the immune response, and antibiotics can disrupt this balance, potentially reducing the efficacy of immunotherapies like checkpoint inhibitors. Studies have shown that antibiotic use prior to or during cancer treatment is associated with poorer responses and reduced survival rates in patients with cancers such as melanoma and lung cancer^3,4^. Additionally, the rise of antibiotic-resistant bacteria poses a significant challenge for cancer patients, who are often immunocompromised and more susceptible to infections. This can lead to delays in treatment, increased morbidity, and higher mortality rates. Addressing antibiotic resistance through stewardship and exploring microbiome-based interventions may therefore improve cancer treatment outcomes.

Breast cancer as one of the most prevalent malignancies among women is specified by uncontrolled proliferation and dissemination of abnormal cancer cells^5,6^. It can progress to the metastatic stage, spreading to distant organs such as the liver, bones, brain, and lungs. Conversely, various treatment options—including radiation therapy, surgery, targeted drugs, chemotherapy, and hormone therapy—have shown no effective results in treating this disease^7–9^. Therefore, it is essential to seek efficient, cost-effective, and biocompatible alternative treatment options. Recently, research has demonstrated the potential of nanoparticles (NPs), particularly AgNPs, in medicine and chemotherapeutics due to the owning the high levels of biological effects such as anticancer^10^, antimicrobial effects. Nanotechnology is a leading field in materials science that effectively integrates various basic science disciplines including engineering^11^, physics^12^, chemistry^13^ and life sciences^14^. The use of nanotechnology in medicine depends on the natural scale of bio-logical phenomena to produce accurate tools for prevention, diagnosis and treatment of disease^15^. NPs have a very high surface-to-volume ratio due to their very small size.

Various mechanical and chemical methods can produce nanomaterials; however, these methods pose environmental risks. As a result, the green synthesis approach has been favored over traditional chemical and physical methods because it is cost-effective, environmentally friendly, and accessible.^16^. Silver nanoparticles exhibit unique physical and chemical properties that render them ideal for various biomedical applications, such as drug delivery and cancer therapy. The incorporation of plant extracts in the synthesis of nanoparticles not only improves their stability and biocompatibility but also introduces bioactive compounds that may work synergistically to combat cancer cells. Previous studies have shown that AgNPs can induce apoptosis in cancer cells, highlighting their potential to enhance the effectiveness of existing treatments^2,17–19^.

AgNPs synthesized from plant sources are not only recognized for their therapeutic potential but also for their antimicrobial properties^20^. This dual functionality makes them particularly advantageous in clinical settings, where infections can complicate cancer treatment. By demonstrating both cytotoxicity against MCF7 cells and antimicrobial activity, AgNPs derived from Anzaroot could serve as multifunctional agents in oncology^21^.

Various parts of plants, such as seeds, roots, leaves, fruits, stems, or even the whole plant extract, can be used to produce different kinds of metal nanoparticles. These extracts contain a wide range of phytochemicals, which act as both reducing and stabilizing agents during the synthesis of nanoparticles. Additionally, the size and shape of the nanoparticles can be finely tuned by adjusting factors like salt concentration, pH levels, and the duration of the reaction^22–26^.

Anzaroot a member of the Fabaceae family, is a valuable medicinal plant known for its compounds—saponins, flavonoids, and polysaccharides—that effectively treat heart diseases, inhibit cancer cell growth, and mitigate chemotherapy side effects^21,27–30^. Certain shrubby Asiatic *Astragalus* species secrete gum tragacanth, a valuable natural substance with high commercial value. It is primarily a high molecular weight hydrophilic carbohydrate polymer with widespread application in the food, pharmaceutical, and cosmetic industries. The green synthesis of AgNPs using the plant Anzaroot has been investigated for its potential cytotoxic effect on MCF7 human breast cancer cells. The study reported that the successfully stable AgNPs in nano-sized. The MTT cell viability assay on MCF-7 cell showed a concentration-dependent inhibition of AgNPs, with an IC50 value of 12.35 μg/mL. Also, Fluorescent microscopic analysis revealed the morphological changes and generating reactive oxygen species (ROS) that modulate oxidative stress in MCF7 cells^21,27–30^.

The MCF7 cell line serves as a well-established model for investigating human breast cancer, allowing scientists to explore the effects of various compounds on tumor growth and proliferation. By assessing the cytotoxicity of silver nanoparticles derived from Anzaroot, this research aims to uncover their potential as effective anticancer agents. Through the use of multiple assays, the study will evaluate cell viability and apoptosis induction, seeking to elucidate the specific mechanisms by which these nanoparticles exert their effects on cancer cells^21,31^.

In the realm of nanomedicine, the use of metal NPs, particularly green synthesized AgNPs as anticancer agents have shown promise. As reported in the previous studies the AgNPs’ IC50 concentrations and the concentration-dependent cytotoxicity of NPs employed at various concentrations was similar. In addition, it has indicated that the cytotoxicity of silver nanoparticles is influenced by their size. More investigations are required due to the limited number of studies in this field, the abundance of phytochemicals in plants, and the unique properties of nanoparticles. Additionally, the research on the cytotoxic effect of AgNPs against cancer cell lines is limited, focusing on some cancer cell lines such as A549 HepG2, MCF-7, HeLa, and U937. As far as our knowledge, this is the first study to assess the anticancer effect of AgNPs using *A. fasciculifolius* Biossplant.

The eco-friendly and cost-efficient green synthesis of AgNPs using Anzaroot leverages natural plant extracts to improve the stability and biocompatibility of the NPs. This method reduces the harmful byproducts typically generated in conventional chemical synthesis while harnessing the bioactive properties of the plant, which could provide additional benefits in targeting cancer cells. However, the process faces certain challenges, such as inconsistencies in NPs size and shape caused by variations in the composition of the plant extracts. Moreover, scaling up this approach for commercial use can be difficult, highlighting the need for detailed characterization of the synthesized nanoparticles to ensure uniformity, quality, and effectiveness in biomedical applications. The study aimed to evaluate the cytotoxic effects of Anzaroot, a medicinal plant native to southeastern Iran, alongside green synthesized AgNPs derived from its aqueous extract, on the MCF-7 breast cancer cell line. Various parameters for optimizing gAgNPs synthesis were assessed, including extract volume, silver nitrate concentration, reaction time, and pH levels. Characterization of the synthesized nanoparticles was conducted using advanced techniques such as Transmission electron microscopy (TEM), Fourier transform infrared (FTIR) spectroscopy, ultraviolet–visible (UV–Vis) spectroscopy, and X-ray diffraction analysis (XRD). This study try to be a bridge the gap between traditional herbal medicine and modern nano-technology. By investigating the green synthesis of AgNPs derived from Anzaroot and assessing their effects on breast cancer cells, it seeks to establish a foundation for future research on plant-derived nanomaterials (NMs) as effective therapeutic agents. The findings may lead to innovative treatments that harness the natural properties of plants to address urgent health challenges like antibiotic resistance and cancer proliferation. The findings are expected to provide insights into the potential of Anzaroot and its nanoparticles in cancer therapy. Ultimately, this research highlights the significance of traditional plants in developing novel anticancer agents. This study distinguishes itselfstands out from previous reports by focusing concentrating on *Astragalus fasci-culifolius*, a lesser-explored less-explored plant species for AgNP synthesis,the synthesis of AgNPs, and evaluating assessing its cytotoxic effects specifically on MCF7 human breast cancer cells. By integrating combining both the synthesis process and with a biological evaluation, it contributes to this research enhances our more comprehensive understanding of plant-derived nanoparticles in cancer therapy, potentially paving the way for innovative therapeutic approaches.

## Materials and methods

### Preparation of aqueous extract of gum and root of* A. fasciculifolius* Bioss

Gum and roots of *A. fasciculifolius* Bioss plant were collected from natural vegetation of Saravan city, Sistan and Baluchestan province, Iran. The collection of *A. fasciculifolius* Bioss plant materials was performed according to institutional, national, and international guidelines. All methods were carried out in accordance with relevant institutional, national, and international guidelines. It was identified by Dr. Yousef Ajani, and maintained in Tehran University of Medical Sciences, Iran (with Herbarium code: PMP-838)) (Supplementary File Fig. S1). In this research, the authors went to Agricultural Jihad Management Organization of Saravan city and has gotten permission to use this genotype from the head of this department.

Collected gum and root of *A. fasciculifolius* Bioss were ground to fine powder, separately. Obtained powders were mixed with Deionized distilled water was added to the obtained powder in 1:100 ratios (powder: water). Then, the mixture was well mixed under stirrer 60 rpm at 30 °C for 60 min. The aqueous extracts were centrifuged at 5000 rpm for 10 min. Finally, the transparent extract was collected and stored at 4°C until use.

### Biosynthesis of AgNPs

First, silver nitrate solutions were prepared at various concentrations (1, 5, and 10 mM). Next, 20 ml of plant extract was sequentially added to 10 ml of the silver nitrate solutions at each concentration. After double distillation, the total volume reached 50 ml. A color change from colorless to brown in the AgNO_3_ solution indicated the biosynthesis of silver nanoparticles (AgNPs). The solution’s absorption intensity was measured using a UV–Vis spectrophotometer (Jenway 6715) shortly thereafter. To ensure accuracy, this procedure was repeated twice^32^.

### Optimization of the factors affecting AgNPs synthesis

In order to produce nanoparticles with a consistent shape and smaller size, different variables influencing AgNPs synthesis, including room temperature, reaction time, extract volume, pH of the reaction solution, and the concentration of silver nitrate solution, were subsequently examined (Supplementary File Table S1).

The investigated *Anz@AgNPsAnz@AgNPs* treatments were as follows: Root extract(R), Gum extract (G), 1mM AgNO_3_ + Root extract (R1); 5mM AgNO_3_ + Root extract (R2); 1mM AgNO_3_ + Gum extract (G1), and 5 mM AgNO_3_ + Gum extract (G2).

#### Optimization of the reaction pH

To optimize the pH of the reaction solution, five series of solutions were prepared, each containing 4 ml of extract and 10 ml of 1 and 5 mM silver (I) salt solutions at pH levels of 2, 4, 6, 8, and 10. The ideal pH level was determined using UV–Vis spectroscopy. To ensure the accuracy of the experiment, this procedure was repeated twice. A 0.1 M solution of either sodium hydroxide or hydrochloric acid was used to adjust the pH of the mixture^33^.

#### Optimization of the extract volume

In order to obtain the best conditions for the synthesis of nanoparticles with the smallest dimensions and the best morphology, the synthesis parameters were optimized. These parameters include 1- Reaction pH 2- Volume of extract consumed 3- Concentration of silver nitrate salt 4- Reaction time 1- Reaction pH: To optimize the reaction pH, 5 series of solutions (2, 4, 6, 8, and 10) were made, including 4 ml of extract and 10 ml of silver nitrate salt with concentrations of 1 and 5 mM. The optimal pH is 8 2- Volume of extract consumed: 4 series of solutions (1 to 4 cc of extract), 10 cc of nitrate salt with concentrations of 1 and 5 mM were made, and the reaction pH is 8. And the volume of extract consumed is 4 cc. 3- Optimal silver nitrate concentration: The volume of extract was optimized and different concentrations (1, 5 and 10) mM silver nitrate and the volume consumed was 10 cc. The pH of the solutions was adjusted to the optimal pH and the UV spectrum was taken from the sample and the optimal silver nitrate concentration was used. 4- Reaction time: We make a sample with all the optimal ones with a duration of (30–60–300) minutes, which was the optimal time.

#### Optimizing the reaction time and studying the stability of SNP

30 min, 60 min, and 300 min after synthesis. All previously adjusted conditions were taken into account to optimize response time and assess the stability of AgNPs. To validate the experiment, this section was repeated twice. After independently analyzing each solution using UV–Vis spectroscopy, the optimal reaction time was determined^33^.

#### AgNO_3_ concentration optimization

The ideal volume of the extract was added to 10 ml of silver nitrate solutions with different concentrations (1, 5, and 10 mM), in order to investigate and optimize the impact of metal ion concentration on nanoparticle synthesis. To confirm the experiment, this section was repeated for two times. The ideal reaction pH was subsequently set. After observing each solution independently using UV–Vis spectroscopy, the ideal silver nitrate concentration was eventually determined ^34^.

### Synthesized AgNPs characterization

The *Anz@AgNPsAnz@AgNPs* were characterized using UV–Vis spectrophotometry (UV-2550, Shimadzu, Japan) and power X-ray diffraction (PXRD; D8-Advance, Bruker, Germany). To finding the size and shape of AgNPs were carried out by transmission electron microscopy (TEM; Zeiss-EM10C-80 kV). Assessment was carried out using 200 kV Ultra Tall Resolution transmission electron magnifying lens (JEOL-2010) where TEM networks were arranged by putting a drop of solution on a carbon-coated copper lattice and drying beneath light. The stabilizing and reducing chemical groups on the surface of nanoparticles were identified using FT-IR spectroscopy (PerkinElmer)^34^. The size of *Anz@AgNPsAnz@AgNPs* was stimated by the De The crystallite average size of the *Anz@AgNPsAnz@AgNPs* has been estimated using Debye Scherrer equation^35^:

1 $$D = \frac{K\gamma }{{\beta Cos\theta }}$$

where K represents the Scherrer constant (0.98), D is the crystallite size, λ is the wavelength of the X–ray source (0.15406 nm), β is the FWHM and θ is the angle of diffraction.

### Cell culture

The human breast cancer cell line MCF-7 was purchased from the Iran University of Medical Sciences’ cell repository, Tehran, Iran. A solution of 1% penicillin–streptomycin (Pen-Strep, Inoclon, and Tehran, IRAN) and 10% fetal bovine serum (FBS, GIBCO, and Grand Island, NY) was added to DMEM (Gibco, Invitrogen Corp.) (PAN-biotech) in all cell treatments. The cells were then maintained at 37 °C under standard conditions including 5% CO_2_ and 95% air humidity. The cells were removed from growth flasks and seeded into a 96-well plate for the subsequent analysis using trypsin-(ethylene diamine tetra acetic acid) EDTA (0.25%, Inoclon, Tehran, IRAN).

#### Cell viability assessment using MTT assay

We followed the well-known 3-(4,5-dimethylthiazol-2-yl)- 2,5-diphenyltetrazolium bromide (MTT) assay to assess the viability of MCF-7 cells (1 × 10^4^ cells/well) exposed to escalating concentrations of 1mM AgNO_3_ + Root extract (R1); 5mM AgNO_3_ + Root extract (R2); 1mM AgNO_3_ + Gum extract (G1), and 5 mM AgNO_3_ + Gum extract (G2), Root extract (R), and Gum extract (G) treatment. In this assay, formation of blue formazan by mitochondrial activity is used to monitor the metabolic activity of MCF-7 cell and determine the cell viability (Formula 1). First, cells (1 × 10 cells/well) were seeded in 96-well microplates and incubated under defined conditions for 24, 48, and 72 h. Next, seven different quantities of Anz@AgNPsAnz@AgNPs(0 to 1000 μg nanoparticle/mL medium) were added to the cells based on serial dilution method (Supplementary File Table S2). Following a period of 4-h exposure to NPs, the cells were cleaned using phosphate-buffered saline (PBS), new medium was introduced to each well, and the plate was kept in the incubator overnight. Finally, the optical density (OD) of the samples was measured using a microplate reader (Stat Fax 2100, Awareness Technology, Inc., USA) at the wavelength of 570 nm. Cell viability was determined by division of OD of the treated sample by that of the control group (untreated cells) as follows (Eq. 1)^36^.

$$Cell\; viability \left( \% \right) = \frac{{OD_{sample} - OD_{medium} }}{{OD_{control} - OD_{medium} }}$$

Then, the morphological alterations of MCF-7 treated with different concentrations of synthetized were qualitatively assessed using inverted microscopy based on cell density^36^.

#### The inhibitory concentration evaluation

The IC50 values of Anz@AgNPsAnz@AgNPs for MCF-7 cell line was estimated separately, using dose response curve data.

Using MTT test the Inhibitory Concentration (IC_50_, the concentration at which 50% of the cells die) was was determined. Cells were treated with synthetized NPs at mentioned concentrations. Survival percentages were evaluated at 24, 48 and 72 h. Determining the IC_50_ values are necessary for evaluating the optimal concentration range for further analysis on each NPs^36^.

### Statistical analysis

The data are presented as Mean ± Standard Deviation (SD). GraphPad Prism V.8 and Statistical Package for the Social Sciences v.18 (SPSS, Inc., Chicago, IL, USA) softwares were used for statistical analysis. One-way and two-way ANOVA tests were used when appropriate. *P* value of < 0.05 was considered statistically significant. All assays were done in triplicates.

## Results

### Photosynthesis and parameter optimization for the synthesis of silver nanoparticles

**Fig. 1:**
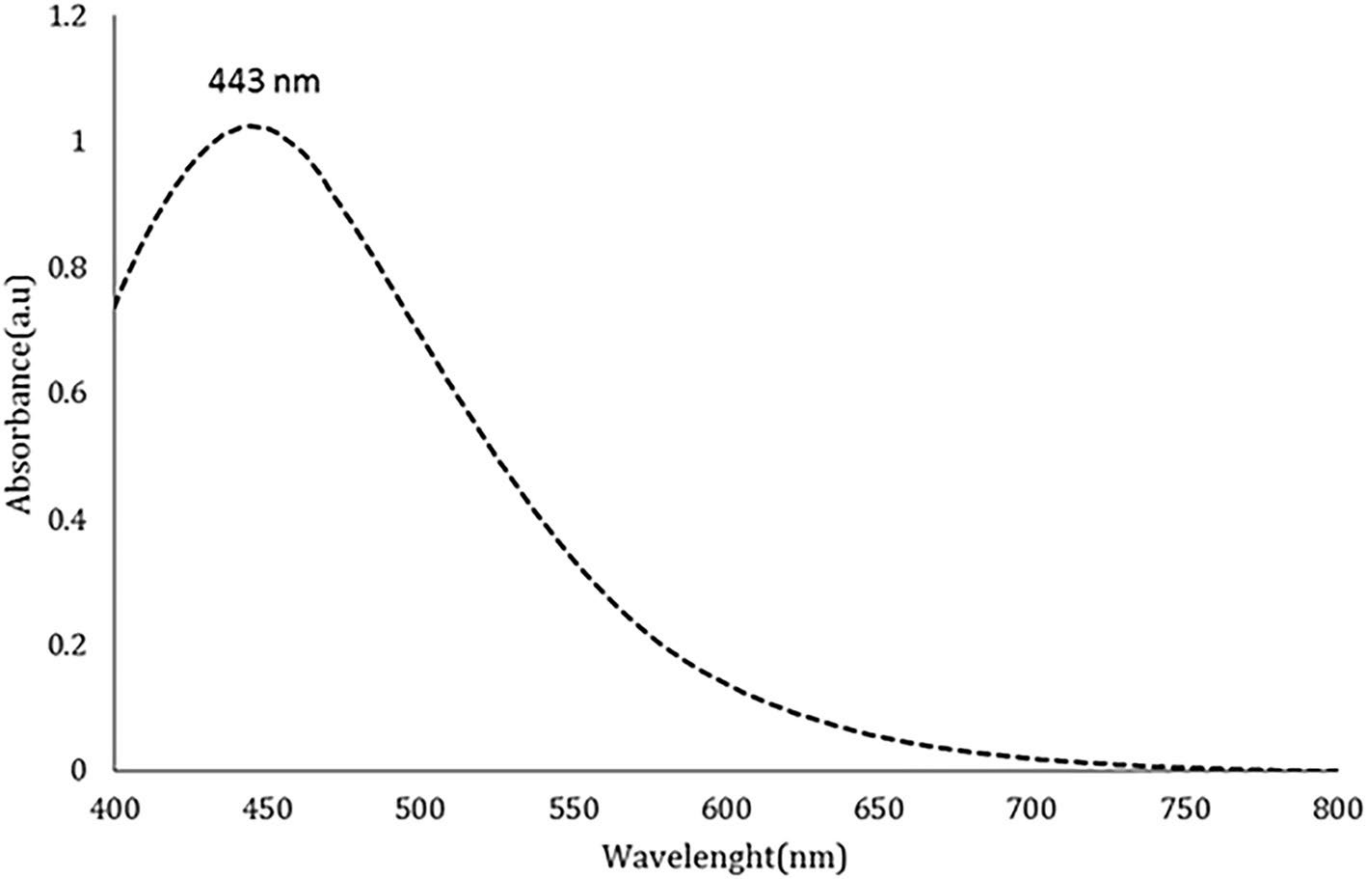
Visible-ultraviolet spectrophotometer spectrum of silver nanoparticles synthesized with *Anzaroot* plant extract.

The reduction of silver ions by plant extracts was monitored using visual characteristics and UV–visible spectroscopy. This study observed a color change from colorless to brown, which served as the initial visual confirmation of silver nanoparticle (SNP) synthesis. Additionally, the presence of a broad absorption band at 443 nm indicated the reduction of silver ions (Ag^+^ to silver nanoparticles (Ag^0^) (Fig. 1).

The following factors influencing AgNPs synthesis were next investigated in order to produce NPs with more uniform morphology and smaller size: reaction time, extract volume, pH of the reaction solution, and concentration of silver nitrate salt.

### pH of the reaction solution

**Fig. 2:**
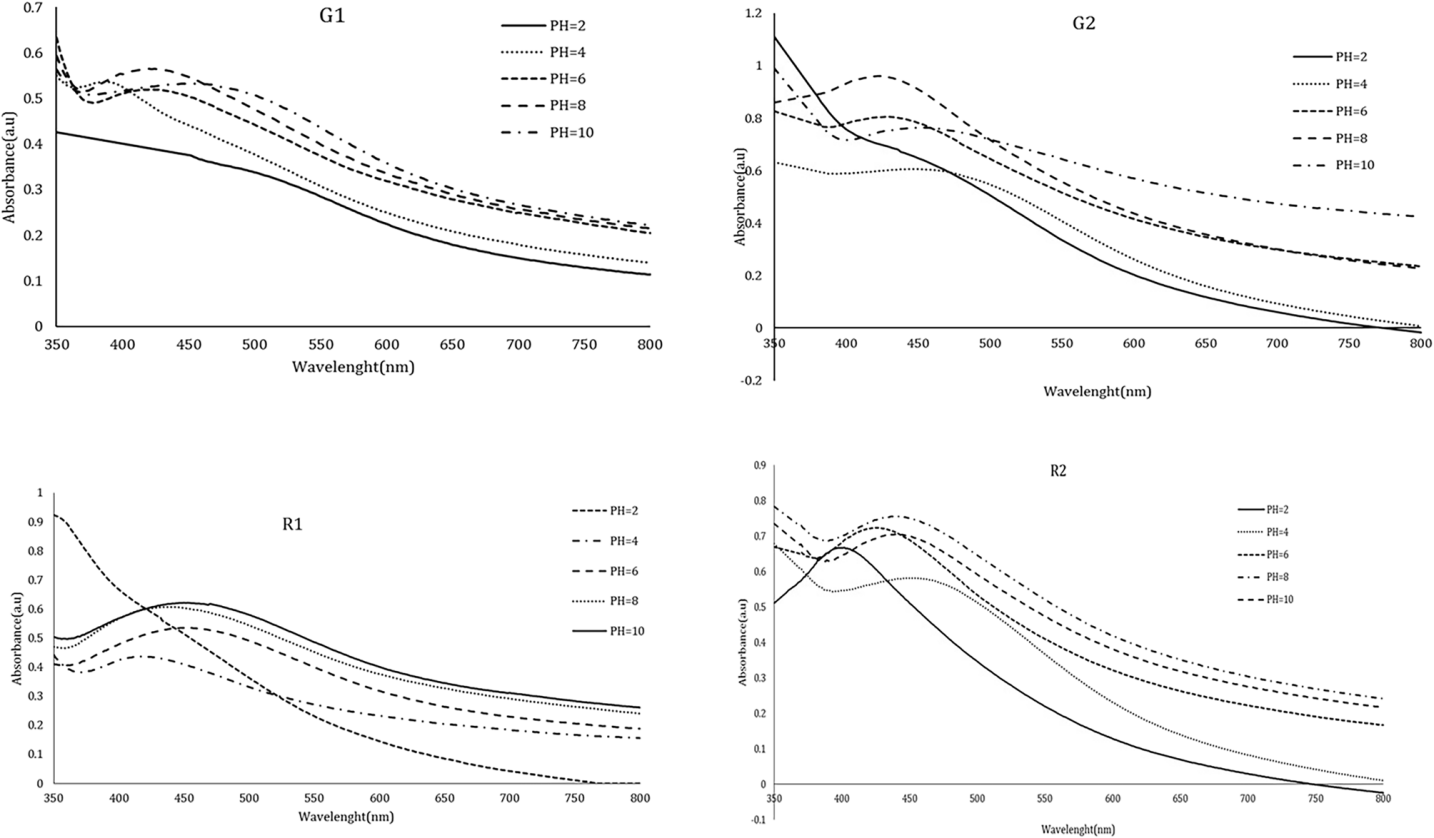
UV–Vis spectrophotometric spectra of the *Anz@AgNPsAnz@AgNPs* synthesized in different pHs from Anzaroot root (R1 and R2) and Gum (G1 and G2).

The synthesis was not conducted at pH 2, as evidenced by the lack of change in absorbance intensity at this pH (Fig. 2). However, when the pH of the solution was gradually increased to 8, there was a dramatic rise in adsorption intensity, corresponding with an increase in SNP production. A distinct symmetric peak was observed at pH 8 in all samples produced from the root and gum extract, indicating that this pH is optimal for synthesis.

### Extract volume

**Fig. 3:**
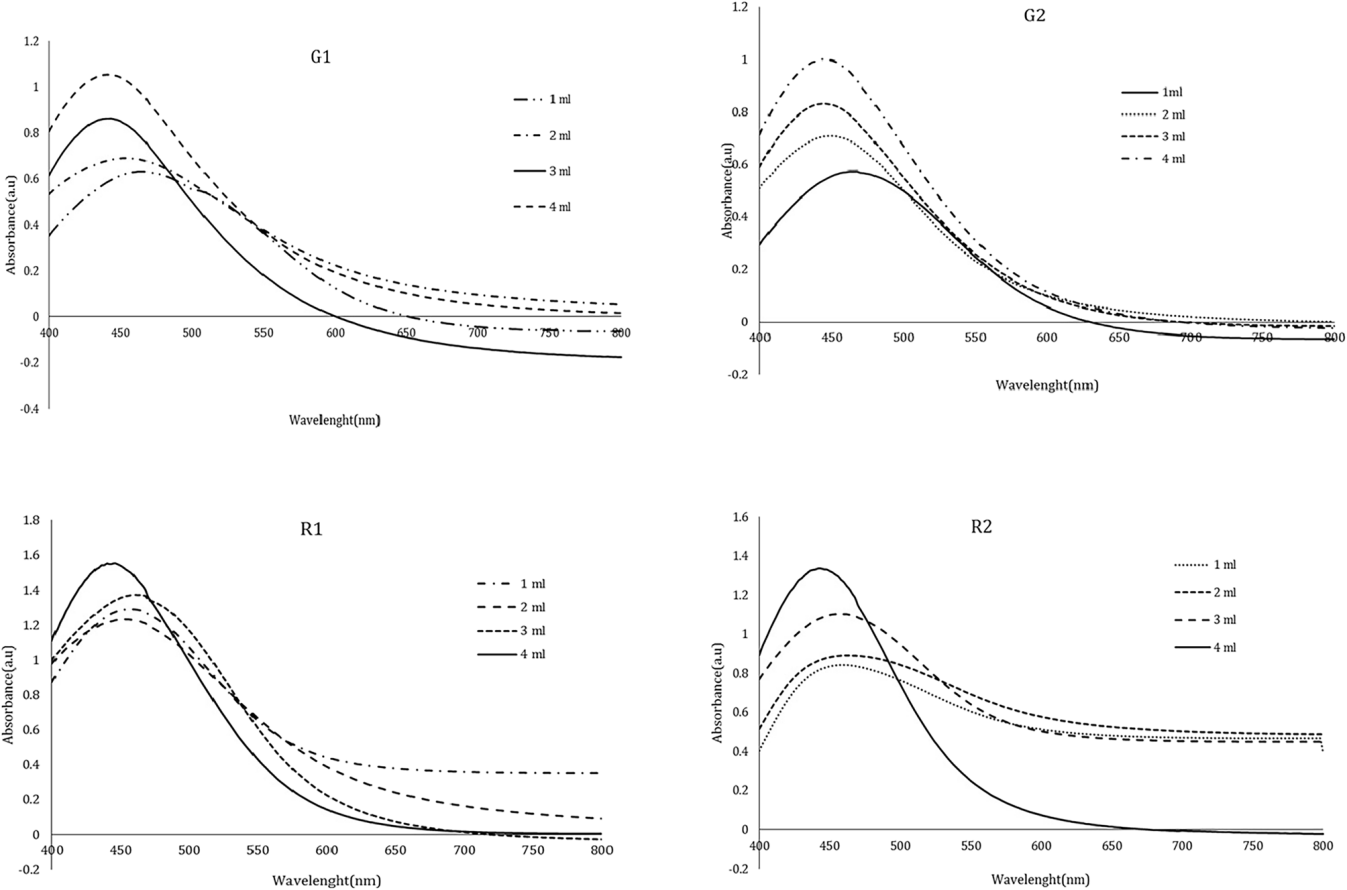
UV–Vis spectrophotometric spectra of the *Anz@AgNPsAnz@AgNPs* synthesized with different valume of the extract from Anzaroot root (R1 and R2) and Gum (G1 and G2).

Investigating the impact of extract volume on nanoparticle synthesis revealed that the strength of the peaks is lower in smaller quantities of plant extract. Furthermore, in-creasing Anzaroot extract concentration promotes peak intensity and absorption intensity, speeds up SNP growth rate, as shown by the SPR red shift in Fig. 3. A direct relation was also found between extract volume and peak intensity so that by increasing the extract volume, the absorbance peak consistently rises as well (Fig. 3). Finally, the 4 ml of root and gum extract was chosen as optimal. With the increase in the amount of the extract, first the maximum absorption intensity related to SPR of the AgNPs increased and then decreased, and also a shift to longer wavelengths was observed.

### Optimizing the concentration of silver nitrate salt solution for silver nanoparticle synthesis

**Fig. 4:**
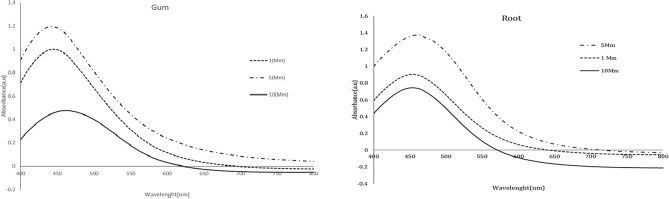
UV–Vis spectrophotometric spectra of the *Anz@AgNPsAnz@AgNPs* synthesized in different concentrations of AgNO_3_.

Using 4 ml of extract, the impact of various AgNO_3_ concentrations (1, 5 and 10 mM) on the formation of silver nanoparticles was examined (Fig. 4). A direct relation was observed between the solution color and AgNO_3_ concentration. Figure 4 shows that the absorption intensity of AgNPs enhanced dramatically when the concentration of silver ions was gradually increased. This rise persisted up to a concentration of 5 mM, after which there was a noticeable drop in NP absorption intensity at a dose of 10 mM. Thus, silver nitrate salt concentrations of 1 and 5 mM were taken into consideration as the ideal concentrations.

### Optimizing the reaction time for silver nanoparticle synthesis

**Fig. 5:**
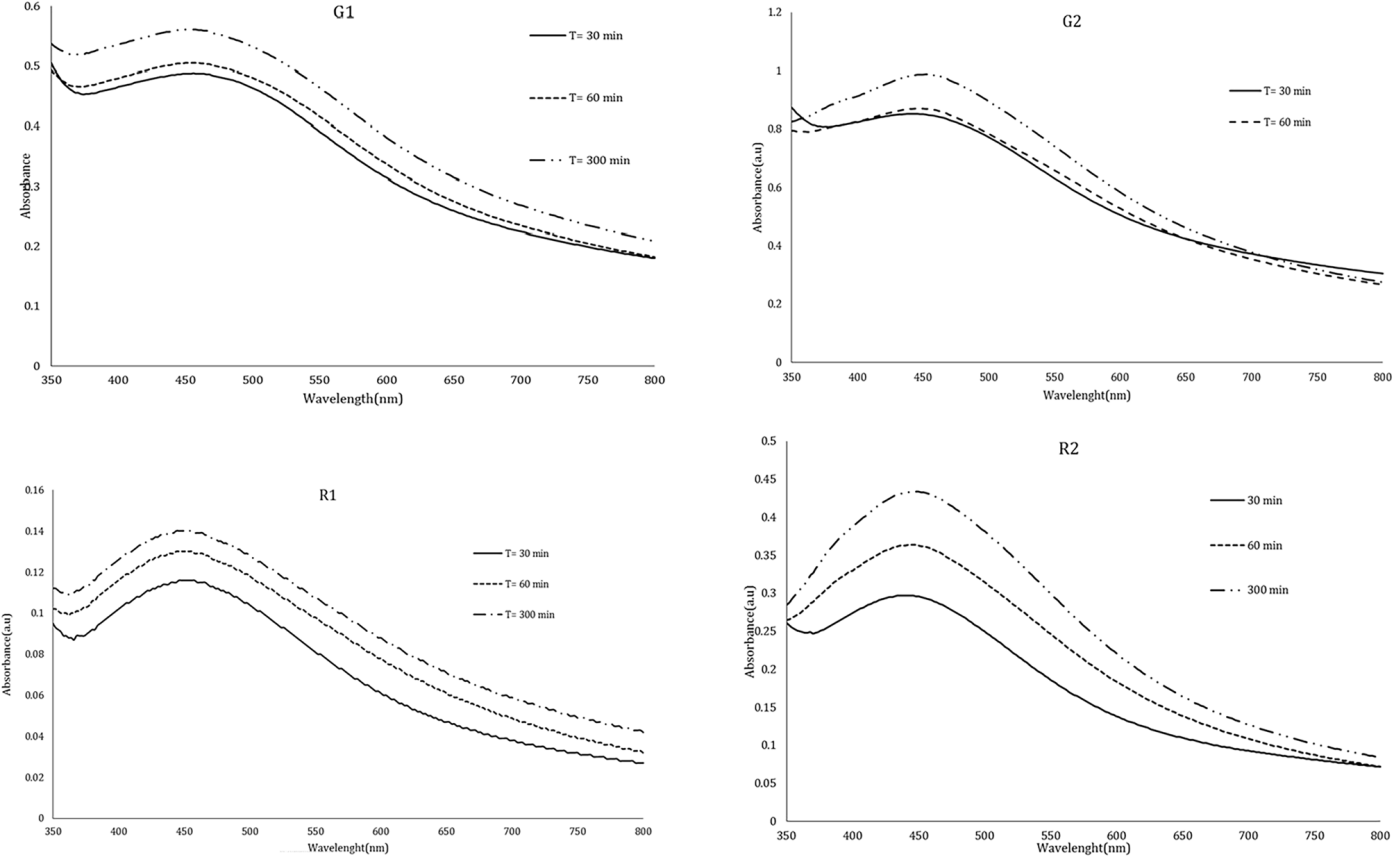
UV–Vis spectrophotometric spectrum in 30, 60, and 300 min from Anzaroot root (R1 and R2) and Gum (G1 and G2).

30, 60, and 300 min (Fig. 5). The intensity of the localized surface plasmon resonance (LSPR) increased over time, peaking around 430–450 nm. Our findings indicated that a longer contact time between the reactants enhanced the absorption. Furthermore, as the reaction time increased, the SPR shifted to a longer wavelength. Therefore, we determined that 300 min was the optimal reaction time.

### Optimum conditions obtained for the synthesis of silver nanoparticles by aqueous plant extract

Finally, to biosynthesis of AgNPs using both the root aqueous extract and the gum aqueous extract of Anzerot plant, pH equal to 8, 4 ml of extract and time of 300 min were obtained as the optimal conditions for the ideal concentrations of ions silver nitrate, 1 and 5 Mm. So, other characterization analyses were documented for the R1, R2, G1, and G2 treatments (Table 1).

**Table 1:** The Uv–Vis maximum absorbance peak of biosynthesized AgNPs using the root and gum aqueous extract of *A. fasciculifolius* Bioss (*Anz@AgNPsAnz@AgNPs*).

		λ_max_ (nm)					
Investigated factor		R1	R2	G1	G2	R	G
pH	2	350	402	350	350	–	–
	4	350	350	350	350	–	–
	6	447	424	350	350	–	–
	8	434	350	350	442	–	–
	10	450	350	350	350	–	–
Extract volume (ml)	1	456	456	460	462	–	–
	2	452	460	452	448	–	–
	3	460	456	440	444	–	–
	4	446	442	440	444	–	–
AgNO_3_ concentration (mM)	1	–	–	–	–	452	400
	5	–	–	–	–	460	442
	10	–	–	–	–	452	465
Reaction time (min)	30	446	434	350	350	–	–
	60	444	442	450	444	–	–
	300	444	444	452	452	–	–

*Treatments: the investigated biosynthesis treatments were as follows: 1mM AgNO_3_ + Root extract (**R1**); 5mM AgNO_3_ + Root extract (**R2**); 1mM AgNO_3_ + Gum extract (**G1**), and 5 mM AgNO_3_ + Gum extract (**G2**).

### Characterization and analysis by FT-IR spectroscopy

The promising biomolecules in the Anzaroot aqueous extract responsible for the reduction of the silver ions and the capping agent responsible for the stability of the bio- reduced AgNPs were identified using FTIR measurements.

**Fig. 6:**
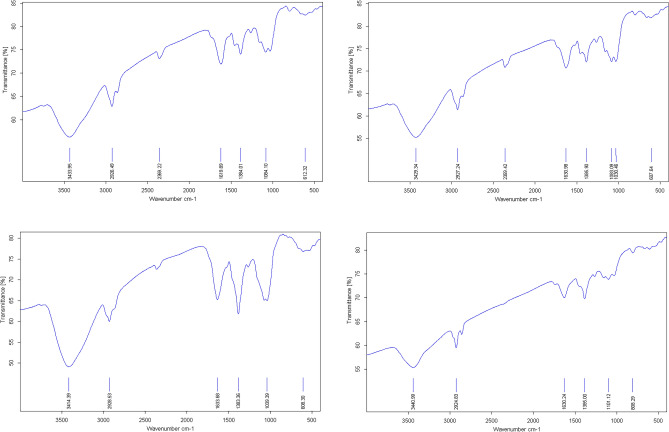
FTIR spectra of the *Anz@AgNPsAnz@AgNPs* synthesized using Anzaroot root (R1 and R2) and Gum (G1 and G2).

**Fig. 7:**
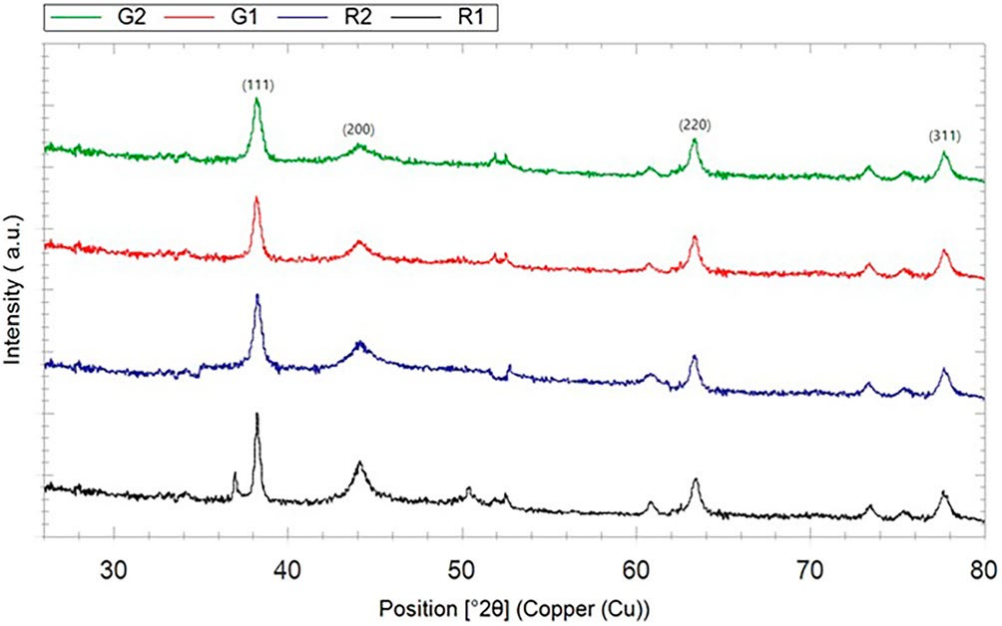
XRD pattern of the *Anz@AgNPsAnz@AgNPs* synthesized with R1, R2 and Gum at G1 and G2.

It has been represented the prominent FTIR peaks of *Anz@AgNPsAnz@AgNPs* synthesized using different plant organs at varying silver nitrate solution concentrations. The FTIR analysis FTIR of AgNPs biosynthesized revealed peaks in the areas of 3400–3500, 2300–2900, 1000–1600, and 600–900 cm^−1^ (Figs. 6 and 7, Table 2). Our results highlighted the stretching vibrations of O–H, C–H, N–H, H–C = O, C = O, C–C, C–O, and C–H bands. These findings verified that Anzaroot secondary metabolites including phenolic and flavonoid chemicals have been successfully encapsulated in AgNPs.

**Table 2:** The observed peaks in FTIR spectrum of biosynthesized AgNPs using the root and gum aqueous extract of *A. fasciculifolius* Bioss (*Anz@AgNPsAnz@AgNPs*) ^37^.

Functional group	Wavelength cm^−1^			
	R1	R2	G1	G2
O–H, amines N–H of amide	3414.39	3440.89	3433.95	3429.34
C–H	2928.53	2924.83	2926.49	2977.24
O = C = O in flavanoids Si–H in silica	–	–	2369.22	2369.42
C–O–H, C = C, C = O	1633.68	1630.24	1619.89	1630.98
C–O in carboxylic acid, and ester	1383.36	1386.00	1384.01	1385.9
C–O–C, N–H, C–N	1039.39	1101.12	1084.01	1083.09, 1030.46
Si–Si, C–Cl, C–Br	–	808.29	–	–
	608.30	–	612.32	607.64

### Characterization by TEM and PXRD techniques

XRD analysis revealed the crystalline structure of Anzaroot AgNPs (Fig. 7). The crystal structure of silver nanoparticles was indicated by four primary peaks at 2θ ranges of 38°, 43°, 64°, and 76°, corresponding to (hkl) values 111, 200, 220, and 311 planes of Ag, respectively, which matched the standard JCPDS No: 01-087-0717. The XRD study has thus confirmed that the *Anz@AgNPsAnz@AgNPs* using the root and gum aqueous extract of *A. fasciculifolius* Bioss having face centered cubic (fcc) crystal structure. Using Scherer’s equation, the average crystal size of bio reduced silver nanoparticles of gum (G1 and G2) and roots (R1 and R2) was calculated as 23.29 nm, 12.4 nm, 15.28 nm, and 12.01 nm, respectively.

The additional peaks observed in the X-ray diffraction (XRD) analysis of *Astragalus fasci-culifolius* Bioss may indicate the presence of distinct crystalline phases or impurities within the synthesized silver nanoparticles. These peaks suggest variations in the crystallinity and size of the nanoparticles, factors that can significantly impact their biological activity and stability. Furthermore, identifying these peaks improves our understanding of the structural properties of AgNPs derived from this plant, which could correlate with their effectiveness against cancer cells.

**Fig. 8:**
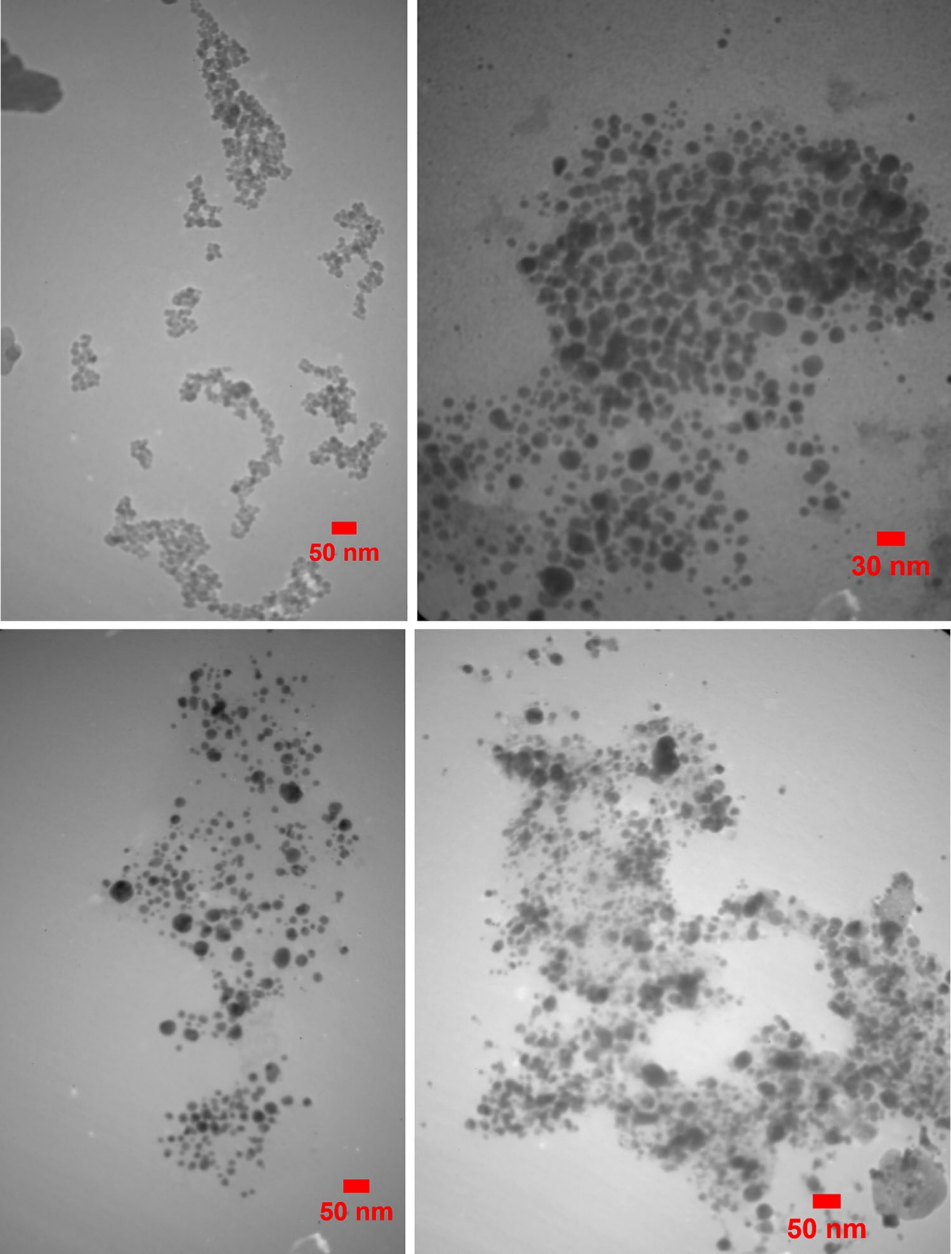
TEM of the *Anz@AgNPsAnz@AgNPs* from root extract (R1 and R2) and the gum extract (G1 and G2).

A TEM examination was conducted, as well in order to determine the morphological properties of the produced AgNPs. The produced AF AgNPs were spherical as well as pseudo-spherical in form (Fig. 8). TEM analysis measured the size of the *Anz@AgNPsAnz@AgNPs* for roots (R1 and R2) and gum (G1 and G2) nanoparticles (Fig. 7).

### Anticancer activity

#### Morphological alterations of AgNPs treated on breast (MCF-7) cancer cells

**Fig. 9:**
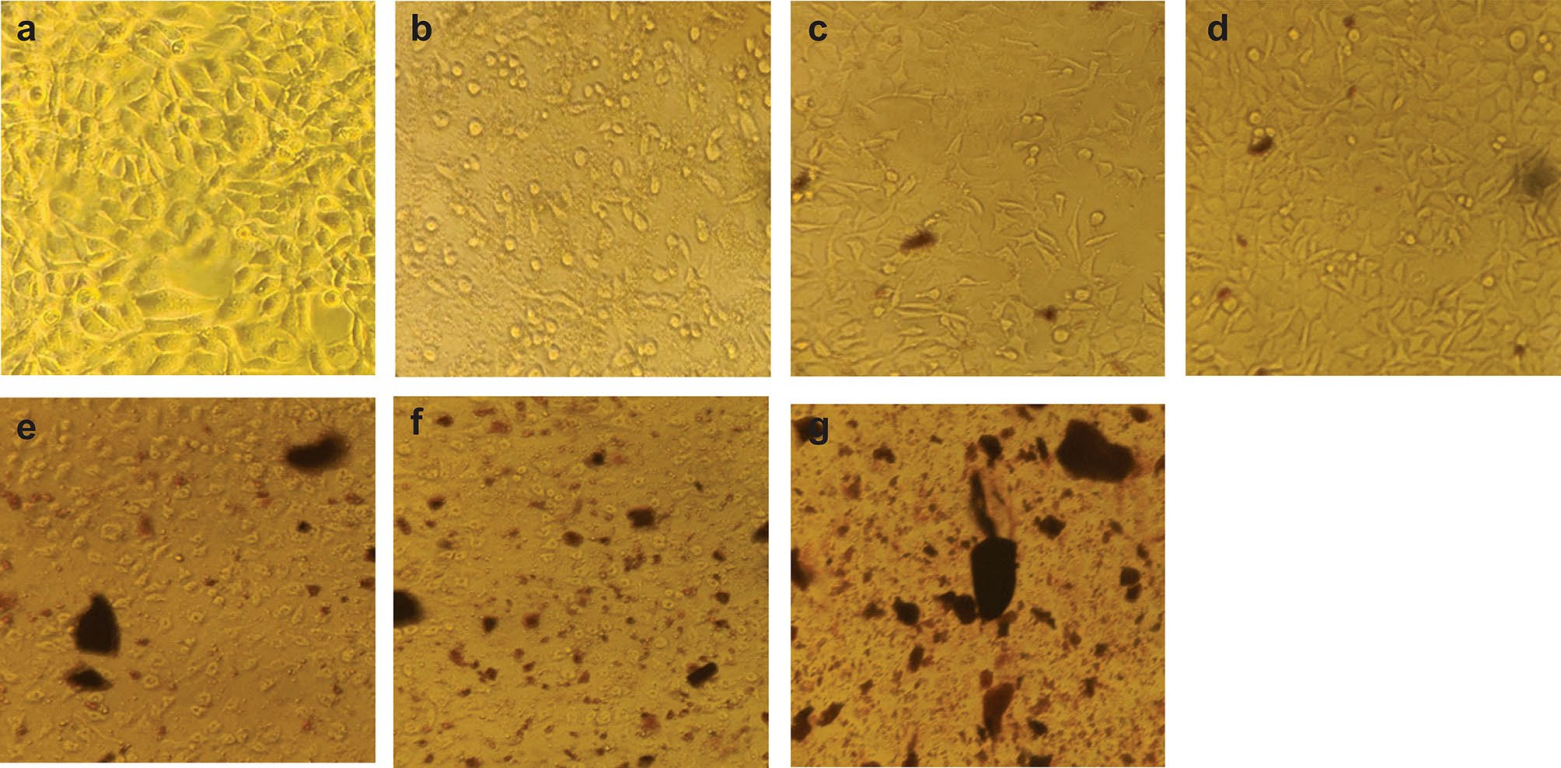
An example Morphological alterations of different concentrations of AgNPs treated on breast (MCF-7) cancer cells (Magnification: 10X). (**a**) control, (**b**) 20 µg/ml, (**c**) 40 µg/ml , (**d**) 80 µg/ml, (**e**): 120 µg/ml, (**f**) 500 µg/ml and (**g**) 1000 µg/ml.

The MTT assay was employed to ascertain the cytotoxicity of Anzaroot extract and its synthesized AgNPs in MCF-7 cancer cell line. It has been shown the percentage of growth inhibition in MCF-7 cell line treated with various Anzaroot extract and nanoparticles dosages (0, 5, 10, 15, 20, 40, 60, 80, 100, 120, 500, and 1000 μg/mL) as compared to untreated cells (Fig. 9). As various amounts of root and gum aqueous extracts were used in order to biosynthesis of the AgNPs, the cytotoxic effect of these nanoparticles was consequently different. Our results showed the anticancer activity of Anzaroot plant at all treatment levels. Hence, Anzaroot R1, R2, G1 and G2 nanoparticles showed cytotoxic effect on MCF-7 breast cancer cell line at dosage of up to 1000 mg/m. Our findings showed that the survival rate of breast cancer cells is decreased with increasing Anzaroot extract and nanoparticles concentrations (Fig. 9).

Our results showed a change in IC50 value with different Anzaroot extract and nanoparticle treatments (Table 3). The IC50 value was measured as 23.50 μg/mL, 21.73 μg/mL, 41.10 μg/mL, 88.63 μg/mL, 384.21 μg/mL and 284.12 μg/mL for R1, R2, G1, G2 nanoparticles and R and G extracts respectively. Our results showed no relation between the IC50 value and the reaction time, so that the IC50 value in all treatments was the same at different time frames (24 h, 48 h and 72 h). The lowest IC50 value among different treatments was belonged to R2 nanoparticle (21.73 μg/mL) and the highest value was belonged to the R extract (348.21) treatment. This 50% cytotoxic concentration was used for further experiments in this study.

**Table 3:** IC50 (µg/ml) of cells treated with plant extract and biosynthesized AgNPs using the root and gum aqueous extract of *A. fasciculifolius* Bioss (*Anz@AgNPsAnz@AgNPs*).

Time	IC50 (µg/ml)					
	R1	R2	G1	G2	R	G
24 h	23.52	21.73	41.103	88.63	384.21	284
48 h	3.94	16.47	36.83	43.25	137.32	85.62
72 h	4.17	9.81	28.43	36.17	46.83	73.49

*Anzaroot Root in R1, R2 and Gum at G1, G2, R and G µg/ml concentration.

#### Effects of AgNPs on cell viability of MCF-7 cells within 24, 48, and 72 h

**Fig. 10:**
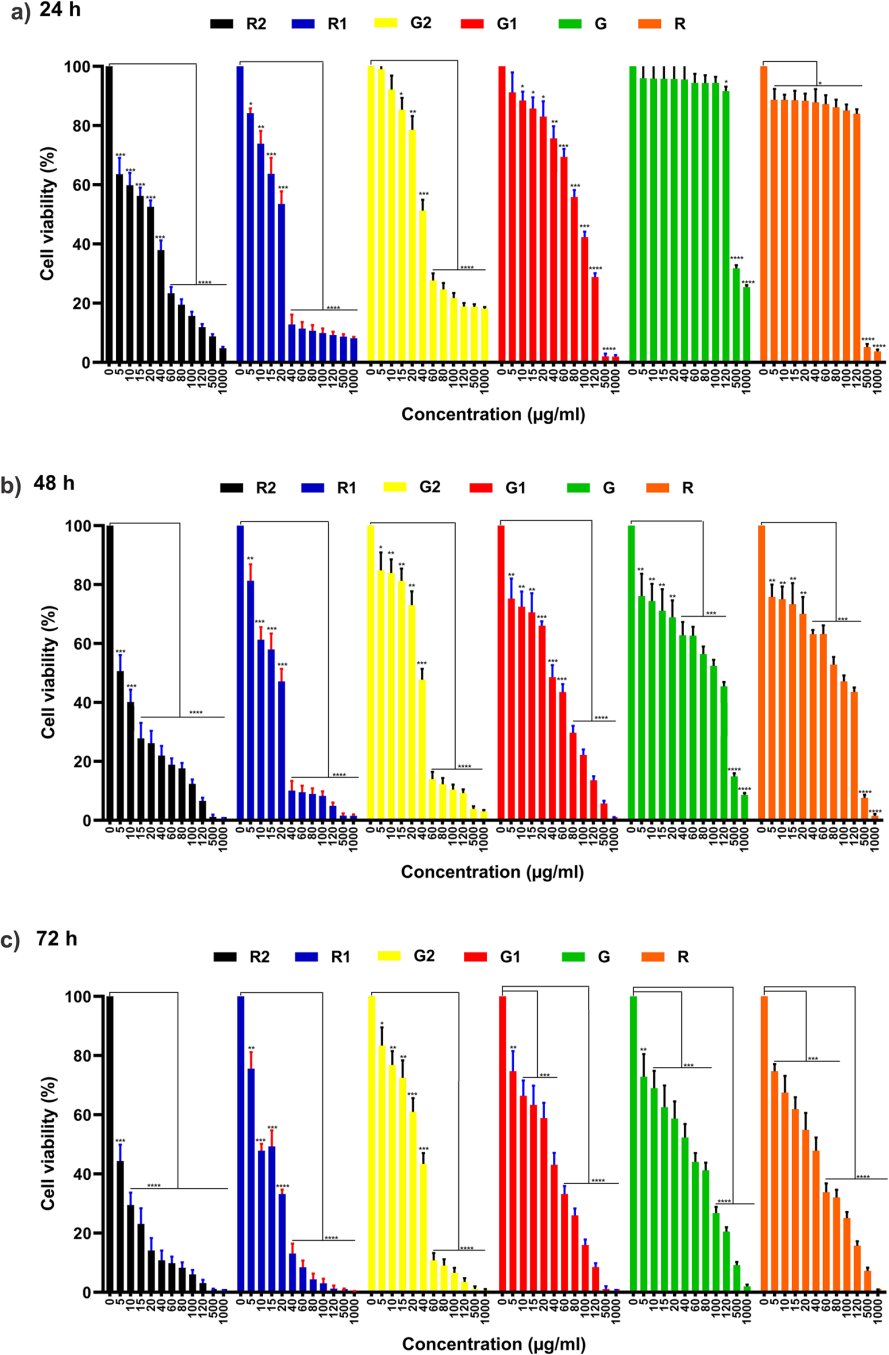
Effects of AgNPs in different concentrations (μg/ml) on cell viability of MCF-7 cells within **a** 24, **b** 48, and **c** 72 hours. (*p < 0.05,**0.01, ***0.001 and ****0.0001 compared to controls).

The results of MTT assay demonstrated the negative effect of Anzaroot extracts and *Anz@AgNPsAnz@AgNPs* on the proliferation ability of MCF-7 cancer cell line in a dose-dependent manner. Additionally, the findings demonstrated that, at comparable doses to the extracts, the nano treatments suppressed cell growth (Fig. 10). Figure 10 illustrates the proliferation rate of the MCF-7 cancer cell line when exposed to various concentrations of AgNP and Anzaroot extract over periods of 24, 48, and 72 h. The percentage of cell survival in the MCF-7 cancer cell line diminished by 1.5, 5, and 7 times, respectively, after treatment for 24, 48, and 72 h. Among the gum nanoparticles (G1 and G2), G2 demonstrated greater effectiveness, while R2 was the most effective among the root nanoparticles after 72 h of treatment at a concentration of 1000 μg/mL (AgNPs and Extracts). Notably, among all the synthesized nanoparticles, R2 exhibited the highest cytotoxic effect on the MCF-7 cancer cell line (Magnification: 10X).

##### Twenty four hour time interval

In this period, the zero concentration serves as a control, meaning no treatment was applied, and its cell viability is considered 100%. In the other concentrations (5, 10, 15, 20, 40, 80, 100, 120, 500, and 1000), cell viability decreased due to the toxicity of nanoparticles, which damage the cell membrane and trigger apoptosis.

1. **R2**: Concentrations of 5, 10, 15, and 20 showed significant differences from the control at a *P* value of 0.001, while concentrations of 60, 80, 100, 120, 500, and 1000 were significant at a *P* value of 0.0001. This indicates that adding nanoparticles in these ranges significantly altered cell viability and reduced cell debris.
2. **R1**: At a concentration of 5, the difference was significant at a *P* value of 0.05; at 10, it was significant at 0.01; at 15 and 20, it was significant at 0.001; and from 40 to 1000, it was significant at 0.0001.
3. **G2**: There was no significant difference at concentrations of 5 and 10. However, at 15, the difference was significant at a *P* value of 0.05; at 20, it was significant at 0.01; at 40, it was significant at 0.001; and from 60 to 1000, it was significant at 0.0001.
4. **G1**: The 5% concentration was not significant, but concentrations of 10, 15, and 20 were significant at a *P* value of 0.05; 40 was significant at 0.01; 60 to 100 was significant at 0.001; and 120, 500, and 1000 were significant at 0.0001.
5. **G**: No significant difference was observed between the control (0 concentration) and concentrations of 5, 10, 15, 20, 40, 60, 80, 100, and 120. However, concentrations of 500 and 1000 were significant at a *P* value of 0.0001.
6. **R**: Concentrations of 0 (control), 5, 10, 15, 20, 40, 60, 80, 100, and 120 were significant at the 5% level, while concentrations of 500 and 1000 were significant at a *P* value of 0.0001.

##### Forty eight hour time interval

1. **R2**: Concentrations of 5 and 10 were significant at a *P* value of 0.001, and concentrations from 15 to 1000 were significant at 0.0001.
2. **R1**: Concentration of 5 was significant at a *P* value of 0.01; concentrations of 10, 15, and 20 were significant at 0.001; and concentrations from 40 to 1000 were significant at 0.0001.
3. **G2**: Concentration of 5 was significant at a *P* value of 0.05; concentrations of 10, 15, and 20 were significant at 0.01; concentration of 40 was significant at 0.001; and concentrations from 60 to 1000 were significant at 0.0001.
4. **G1**: Concentrations of 5, 10, and 15 were significant at a *P* value of 0.01; concentrations of 20 and 40 were significant at 0.001; and concentrations from 80 to 1000 were significant at 0.0001.
5. **G**: Concentrations of 5, 10, 15, and 20 were significant at a *P* value of 0.01; concentrations from 40 to 120 were significant at 0.001; and concentrations from 500 to 1000 were significant at 0.0001.
6. **R**: Concentrations of 5, 10, 15, and 20 were significant at a *P* value of 0.01; concentrations from 40 to 120 were significant at 0.001; and concentrations from 500 to 1000 were significant at 0.0001.

##### Seventy two hour time period

1. **R2**: Concentration of 5 was significant at a *P* value of 0.001, and concentrations from 10 to 1000 were significant at 0.0001.
2. **R1**: Concentration of 5 was significant at a *P* value of 0.01; concentrations of 10 and 15 were significant at 0.001; and concentrations from 20 to 1000 were significant at 0.0001.
3. **G2**: Concentration of 5 was significant at a *P* value of 0.05; concentrations of 10 and 15 were significant at 0.01; concentrations of 20 and 40 were significant at 0.001; and concentrations from 60 to 1000 were significant at 0.0001.
4. **G1**: Concentration of 5 was significant at a *P* value of 0.01; concentrations of 10, 15, 20, and 40 were significant at 0.001; and concentrations from 60 to 1000 were significant at 0.0001.
5. **G**: Concentration of 5 was significant at a *P* value of 0.01; concentrations of 10, 15, 20, 40, 60, and 80 were significant at 0.001; and concentrations from 100 to 1000 were significant at 0.0001.
6. **R**: Concentrations of 5, 10, 15, 20, and 40 were significant at a *P* value of 0.001, and concentrations from 60 to 1000 were significant at 0.0001.

## Discussion

The synthesis of silver nanoparticles (AgNPs) using plant extracts requires a thorough analysis of phytochemicals through both quantitative and qualitative methods. Quantitatively, optimal conditions for SNP synthesis were established, including a plant extract volume of 4 ml, silver nitrate concentrations of 1 mM and 5 mM, a reaction time of 300 min, and a pH level of 8. These conditions correlated with enhanced absorption intensity observed in UV–visible spectroscopy. Qualitative analysis, conducted using FTIR spectroscopy, identified functional groups responsible for reducing silver ions and stabilizing AgNPs, such as O–H, C–H, and C = O bonds. Additionally, secondary metabolites like phenolic compounds and flavonoids were found encapsulated within the nanoparticles, contributing to their antioxidant properties. X-ray diffraction (XRD) confirmed that the AgNPs have a face-centered cubic structure, with sizes ranging from 12.01 nm to 23.29 nm. Cytotoxicity tests using the MTT assay demonstrated varying effects on MCF-7 cancer cells, with R2 nanoparticles exhibiting the highest cytotoxicity, achieving an IC50 value of 21.73 µg/mL, indicating significant potential for anticancer applications.

The MTT assay was used to assess the cytotoxicity of Anzaroot extract and its synthesized silver nanoparticles (AgNPs) on the MCF-7 cancer cell line, owing to its reliability in measuring cell viability. This assay relies on the ability of metabolically active cells to reduce MTT, a yellow tetrazole, to purple formazan crystals, which enables the quantification of cell proliferation and viability. By evaluating the percentage of growth inhibition at different dosages, the MTT assay offers valuable insights into the anticancer potential of Anzaroot extracts and AgNPs, allowing for comparisons with untreated control cells^37–39^.The green synthesis of AgNPs from plants has shown promising cytotoxic effects on MCF7 human breast cancer cells in vitro. Studies have demonstrated that biosynthesized AgNPs (AgNPs) from plant sources exhibit significant efficacy against cancer cells, particularly MCF7 cells. These AgNPs have been found to have cytotoxic activities against MCF-7 cell lines, indicating their potential as alternative cytotoxic agents. The cytotoxicity of AgNPs is influenced by their size and shape, with specific sizes and shapes showing potential cytotoxic effects on cancer cells. Additionally, AgNPs synthesized via plant sources have displayed significant cytotoxic effects on MCF-7 breast cancer cell lines, suggesting their potential as antitumor agents in the treatment of metastatic breast cancer. The mechanism of action of these AgNPs involves interactions with cancer cells, leading to DNA damage, mitochondrial dysfunction, apoptosis activation, and inhibition of cell proliferation^40^.

Biological synthesis is widely regarded as the most suitable technique for nanoparticle production compared to chemical and physical methods. While physical and chemical nanoparticle synthesis techniques can be efficient, they have notable drawbacks, including high costs, significant energy requirements, and the generation of hazardous and toxic waste. Additionally, it has been observed that some hazardous substances can adhere to chemically produced nanoparticles, rendering them unsuitable for biological applications. In this study, during the production of silver nanoparticles (AgNPs), the solution color changed from white to brown upon mixing 4 ml of Anzaroot extract with 10 ml of silver nitrate solution at concentrations of 1 and 5 mM. This color change indicates successful nanoparticle synthesis. Several factors, such as particle size, shape, interactions, and refractive index, can influence the optical absorption spectrum of metallic nanoparticles. The shape of nanoparticles not only affects their optical and electrical properties but is also a critical factor in determining their potential applications. The observed brown color can be attributed to surface plasmons, which arise from the collective oscillations of valence electrons in the electromagnetic field of incident radiation. The UV–vis spectra of the _Anz@_AgNPs displayed a plasmon resonance at 400 nm. Typically, the maximum wavelength (λ max) of AgNPs ranges from 400 to 500 nm. The position and shape of the surface plasmon absorption bands depend on the shape and size of the synthesized particles, their interparticle distance, and the dielectric constant of the surrounding medium. Consistent with previous findings, another indicator of effective nanoparticle production in our study is the presence of strong peaks in the UV–vis spectrum, with maxima centered between 425 and 440 nm. These peaks align with the surface plasmon resonance commonly exhibited by AgNPs^41–45^.

The synthesis was not performed at pH 2, as the UV–Vis spectra did not show a maximum absorption peak at this pH. However, as the solution’s pH was gradually increased to 8, the maximum adsorption peak rose dramatically, correlating with an increase in the production of silver nanoparticles (AgNPs). In the UV–Vis absorption spectrum, the intensity decreased at a higher pH (pH 10), indicating that the entry of silver ions into the reduction process diminished. At this pH, silver ions are hydrolyzed to produce stable silver hydroxide species. It has been demonstrated that the reaction pH significantly affects the size of the _Anz@_AgNPs. As the overall charge of the clusters reduces the likelihood of particle aggregation, the increased electrostatic and electrosteric repulsive interactions enhance the stability of the cluster dispersion and colloid formation in the alkaline pH range, resulting in the formation of small, monodispersed, spherical nanoparticles with a higher silver content^46^. This is probably because at high pH levels, the nucleation processes and the crystallization of smaller particles from Ag nuclei occur at higher reaction rate. The size and form of AgNPs are influenced by several variables^47^. The size and form of AgNPs are influenced by several variables. It has shown that the peak widens and the absorption intensity increases with increase in pH, which indicates an increase in particle size^48^. As a distinct symmetric peak was noted at pH 8, this pH was determined as the ideal pH in our study. The absorbance intensity was raised with increasing pH level, resulting in a narrow peak with a homogeneous particle size distribution. Therefore, we presume that the fundamental circumstances for controlling particle size were favorable in our study design. Several studies have shown the inverse relation between pH level and silver nanoparticle size, as well^49–51^.

The assessment of the effect of extract volume on nanoparticle production revealed that smaller volumes of plant extract resulted in lower peak strength. This may be attributed to a reduced presence of reducing agents, which slows the rate at which Ag^+^ ions are converted to Ag0. Conversely, increasing the extract concentration enhanced both peak strength and absorption intensity, likely due to a greater quantity and accumulation of silver nanoparticles (AgNPs). Variations in absorption intensity may indicate changes in the size of the nanoparticles. Furthermore, the evaluation of the optimal extract concentration demonstrated that increasing the extract concentration significantly boosts nanoparticle production. Larger volumes of Anzaroot extract accelerated the growth rate, leading to the formation of larger particles, as evidenced by a red shift in the surface plasmon resonance (SPR) spectrum. Overall, a higher extract volume results in more organic material surrounding the nanoparticles, which can cause the distinctive peak of the nanoparticles to diminish in the target range due to particle settling, while still maintaining a high quantity in the nano state. Additionally, the study on the green synthesis of AgNPs using Vaccinium arctostaphylos extract and the assessment of its antibacterial properties confirmed the positive impact of extract volume on nanoparticle synthesis as well^52–55^.

The evaluation of antibacterial and anticancer activity of AgNPs synthesized using leaves of Caesalpinia gilliesii (Hook) also supports our findings, showing a direct correlation between peak intensity and extract volume. Notably, the ratio of extract aliquot to the amount of silver precursor significantly influences the formation of uniformly shaped and well-dispersed AgNPs^56–58^.

Our results showed that the absorption intensity of AgNPs is dramatically increased by the gradual raise in silver ions concentration. This rise persisted up to a concentration of 5 mM, after which there was a noticeable drop in NP absorption intensity at the dose of 10 mM. It was previously reported that synthesis of bigger sized nanoparticles and the binding of NPs cause the reduction of NPs absorption by the rise in metal ion concentration. Thus, silver nitrate solution concentration of 1 and 5 mM was considered as the ideal salt concentration. However, raising the silver nitrate solution’s (colloidal solution) concentration has also accelerated nanoparticle production. Increasing the synthesis of AgNPs is indicated by the intensity of solution color change caused by variations in the concentration of the silver nitrate solution (colloidal solution), which is due to the growth of the particle size. The results of our spectroscopic analysis demonstrated that increasing silver nitrate solution concentration, increases the particle size and, consequently, the peak intensity, as well. However, a further increase in the concentration of silver ions shows an opposite trend. Consistent with the results of other studies, our results showed an increase in AgNPs synthesis rate by increasing the concentration of silver nitrate solution (colloidal solution)). This consequently promotes nanoparticle size and maximum absorption rate, as well^34,59–61^.

UV–vis absorption spectra indicated that the intensity of the LSPR absorption peak increased over time, with the maximum peak observed in the range of 400–500 nm. This clearly demonstrates how free electron transitions lead to the reduction of silver ions to AgNPs. Furthermore, the presence and formation of nearly spherical AgNPs are linked to the LSPR absorption peak. Time, like the other factors mentioned, significantly impacts the stability and synthesis of nanoparticles. Our findings revealed a direct relationship between the duration of reactant contact and the absorption rate. Additionally, the SPR shifted to a higher wavelength with increased reaction time, consistent with the results of previous studies^62–64^.

FT-IR analysis revealed that the oxidation of polyol phenolic components in conjunction with the reduction of Ag^+^ ions may be the plausible process involved in AgNPs synthesis. The FTIR spectra of the *Anz@AgNPsAnz@AgNPs* at varying silver nitrate concentrations and plant organ types revealed peaks in the ranges of 3400–3500, 2300–2900, 1000–1600, and 600–900 cm^−1^ which correspond to some functional groups such as O–H, C–H, N–H, C–O, C =  = O, and etc. That associated with the functional groups of phytochemical constituents in the root or gum of Anzaroot extract capping the Anz@AgNPsAnz@AgNPs^65–67^.

The crystal structure of AgNPs was indicated by four primary peaks at 2θ ranges of 38°, 43°, 64°, and 76°, corresponding to 111, 200, 220, and 311 planes of Ag, respectively. As the same results were reported for another specious of *Astragalus*. Sharifi-Rad and couthers (2020) observed to have identical XRD peaks for AgNPs using *Astragalus tribuloides*. The presence of low-intensity peaks may be attributed to the crystallization of bioorganic phases on the surface of the AgNPs^68^.

The results of TEM analysis showed that the particles are spherical and pseudo-spherical in shape. So, the natural sources including biochemical could be applied for NPs synthesis as reported in other reporters. They showed the biochemical ability in the reduction of Ag^+^ ions to AgNPs^40^. Our measurements using UV–Vis spectroscopy coincide with XRD and TEM imaging. The XRD and TEM results revealed the relation between AgNO_3_ concentration and nanoparticle size which is in line with the findings of previous other studies. In a study the anticancer effect of the *AgNPs* from *Fagonia indica* against MCF-7 cancer cells was investigated. These reported *AgNPs* were spherical with a size range of 2.5, to 13.5 nm associated the varying AgNO_3_ concentrations.

We demonstrated that AgNPs with different cytotoxic activity can be biosynthesized from varying amounts of Anzaroot root and gum aqueous extracts. In addition, the cytotoxic effect of Anzaroot extract and *Anz@AgNPsAnz@AgNPs* (R, G, R1, R2, G1, and G2) against breast cancer cells at up to a concentration of 1000 mg/ml showed the highest anticancer effect for R2. Similar findings have been reported by other study groups. There was no change in the IC50 value throughout the treatment course of 24 h, 48 h, and 72 h. Additional tests in this investigation were conducted using this 50% lethal concentration. Also, our results on the MTT assay demonstrated that both the extract of *A. fasciculifolius* Bioss and *Anz@AgNPsAnz@AgNPs* can reduce the growth rate of the MCF-7 cancer cell line in a dose dependent manner^39,69–72^.

It has been investigated the anticancer potential of AgNPs using Lantana camara leaf extract, on the MCF-7 cells line. The results showed that AgNPs damaged the polymer components of the cell membrane and had a significant effect on the rupture of the cell wall. As the AgNPs concentration increased, the permeability of the membrane increased^73^. It is also reported that an environmentally friendly and safe agent for the synthesis of physiologically active AgNPs was the aqueous extract of red and yellow *Astragalus sarcocolla* gum. Using *A. sarcocolla* gum at different aqueous extract concentrations of 5, 10, and 20 mg/ml (w/v), six AgNPs including three red gum (RG1, RG2, RG3) and three yellow gum (YG1, YG2, YG3) nanoparticles with distinct features were synthesized. Then, the cytotoxic impact of A. sarcocolla gum-AgNPs on five human cancer cell lines was assessed using the MTTassay, as compared to the control. With an IC50 value of 72.2 μg/ml, the semi-rounded particles, which were generated from 5 mg/ml red gum (AgNPs-RG1) and had a size range of 9.23 ± 3.66 nm, showed the highest cytotoxic impact against Mcf-7 cancer cell line^74–77^.

In this study, the growth inhibition of cells treated with AgNP was more than cells treated with the extract at all doses. In our investigation, the IC50 value for *Anz@AgNPsAnz@AgNPs* through root (R) and gum (G) extract synthesized nanoparticles (R1, R2, G1, G2) was measured as 384.21 μg/mL, 284.12 μg/mL, 23.50 μg/mL, 21.73 μg/mL, 41.10 μg/mL and 88.63 μg/mL, respectively. Consequently, R2 nanoparticle with the IC50 value of 21.73 μg/mL and R extract with the IC50 value of 348.21μg/mL had the lowest and the highest IC50 value among all treatments. There was no change in the IC50 value throughout the treatment course of 24 h, 48 h, and 72 h. Additional tests in this investigation were conducted using this 50% lethal concentration. According to our results, different factors including the extract concentration and the type of plant organ used for extraction affects the cytotoxic potential of the AgNPs and the survival rate of breast cancer cells. Also, in the comparison of the investigated treatments, the inhibition rate of cancer cell growth by Anz@AgNPsAnz@AgNPs was shown as R2 > R1 > G2 > G1, while the extracts were ranked as R > G. Therefore, root extract exhibits greater anti-cancer activity than gum extract. Numerous herbs belonging to the Astragalaceae family have shown the ability to suppress cancer cells^78–81^. Furthermore, it has been established that flavonoids derived from *Astragalus complanatus* are significant in suppressing tumor development by inducing human hepatocellular carcinoma cells to undergo apoptosis through the death-receptor-dependent and mitochondria-dependent apoptotic pathways. Numerous investigations on the phytochemistry of the *Astragalus* genus revealed the presence of sterols, mucilages, nitro compounds, saponins, and phenolic compounds. Natural antioxidants are phenolic compounds that are prevalent in nature, in a variety of plant organs, including fruits, vegetables, and roots and leaves. It has been investigated that the cytotoxic effects of the extracts from *A. argaeus* subterranean and aerial sections against MCF-7 cancer cellsand fibroblast cells. MCF-7 and fibroblast cells were treated with extracts at different concentrations of 10, 25, 50, 100, 200, and 400 μg/ml for 24 and 48 h. The percentage of cell viability for A. argaeus root and shoot extracts was measured as 67.19% and 77.06%, respectively, when MCF-7 cancer cells were treated for 24 h at a dose of 25μg/ml. On the other hand, no impact on growth was observed, when MCF-7 cells were treated for 48 h with different doses of subterranean extract^28,82–84^. The percentage of cell viability was measured as 87.34%, following 48 h’ treatment of cells with shoot extract at the concentration of 25 μg/ml. The strong anti-cancer activity of Astragali radix extract has been demonstrated in a study. Our results on the MTT assay demonstrated that both the extract and AgNPs of *A. fasciculifolius* Bioss can reduce the growth rate of the MCF-7 cancer cell line in a dose-dependent manner. Conversely, the spherical to cubic shaped A.sarcocolla gum-AgNPs (AgNPs-YG1) extracted from 5 mg/ml yellow gum showed a strong cytotoxic activity against MCF-7 breast cancer cell line. These nanoparticles were 10.92 ± 5.48 nm, with an IC50 value of 82.8 ug/ml. These findings suggest that there are important biological uses for the produced AgNPs made from tiny amounts of *A. sarcocolla* gum. Investigating the cytotoxic effect of *Astragalus elongatus* and other species of the plant against cancer cells has shown that the root extracts of these plants are more effective as compared to shoot extract. The effects of *Astragalus ovinus* ethanolic extract and its polysaccharides fraction on the anti-proliferation and apoptosis of MCF-7 breast cancer cell line have been studied. The cytotoxicity data showed that following exposure to the polysaccharides fraction or plant extract, the percentage of growth inhibition in MCF-7 cancer cells is increased in a dose-dependent manner. Furthermore, the results of MTT assay showed a remarkable rise in the percentage of growth inhibition of cancer cells at polysaccharide fraction concentration of higher than 400 μg\mL^85^. *Astragalus ovinus* extract showed anti-cancer effects against MCF-7 cancer cells, at a concentration lower than that of the polysaccharides fraction^86,87^. The IC50 value for *Astragalus ovinus* extract, polysaccharides fraction, and cisplatin treatment were measured as 22.42, 560.9, and 961.2 μg/mL, respectively^87–89^.

Our findings indicate that Anz@AgNPsAnz@AgNPs can effectively suppress cancer cell growth at doses comparable to those of plant extracts. Among the various treatments, nanoparticles synthesized from root extracts (R2 and R1) exhibited the highest anticancer activity. The enhanced cytotoxicity of Anz@AgNPsAnz@AgNPs can be attributed to their unique properties, including a high surface-to-volume ratio and superior permeability into cell membranes compared to plant extracts. Their small size facilitates easier entry into cells through endocytosis. Once inside, these nanoparticles can disrupt cellular protein function, altering the structure and chemistry of the cell. Additionally, their small and non-aggregated nature allows them to generate reactive oxygen species (ROS) within the cells^90,91^.

It has been synthesized and investigated the bioactivity of AgNPs from the leaf extract of Ginkgo biloba (Gb), having abundant flavonoid compounds^92^. They concluded that ROS can induce oxidative stress by damaging DNA and altering cell morphology, ultimately resulting in apoptotic cell death. Generally, Anz@AgNPsAnz@AgNPs are a non-toxic, cost-effective, and efficient method for antibacterial and anticancer activity. Programmed cell death or apoptosis occurs when cells undergo stress due to infections, DNA damage, or growth factor deficiencies. This process primarily involves intrinsic mechanisms within the cytoplasm and mitochondria^92,93^. In addition, the other researchers have recently indicated that apoptosis can occur through structural or biochemical changes that lead to cell inactivity, shrinkage, membrane rupture, or DNA fragmentation. Cancer cells exhibit higher permeability and retention, allowing for preferential interaction with SNPs due to their nanoscale size^40^. Continuous entry of AgNPs into target cells disrupts uncontrolled cell division, resulting in cell death. Additionally, tumor cells undergo early apoptosis by regulating physiological factors that control rapid cell division^94,95^. Also, ROS are critical in the apoptosis pathway, weakening membrane permeability and facilitating the release of cytochrome c and procaspases-2 and -3. The combination of cytotoxic and genotoxic agents activates caspases, leading to mitochondrial destabilization. AgNPs respond to intracellular signaling through ROS activation, and p53-mediated apoptosis is notably effective when using AgNPs^96,97^. Cytochrome C, essential for ATP production, triggers nuclear changes, DNA fragmentation, and cellular alterations. The primary mechanism by which AgNPs induce apoptosis in A549 and MCF7 cells is through processes occurring within these cells^98^.

Research on the phytochemical properties of *Astragalus* species has been relatively limited. However, it has been conducted^99^ a study using gas chromatography-mass spectrometry (GC–MS) to analyze the volatile compounds in the aerial parts of six *Astragalus* species. This investigation identified a total of 97 metabolites. Notably, Sylvestrene was the dominant component in A. sieversianus, A. mucidus, and A. macronyx, with the highest concentration found in A. sieversianus at 64.64%. Additionally, (E)-2-hexenal was detected at high levels in A. lehmannianus (97.9%) and A. chiwensis (10.1%). In another study, it has been analyzed^100^ the essential oil from the root of *Astragalus chrysostachys* Boiss. using GC–MS. They identified eight compounds, including Linalool (2.4%), m-Tolualdehyde (29.7%), Undecane (0.4%), Hexahydrofarnesyl acetone (3.7%), acetophenone (16.2%), Carotol (1.5%), Croweacin (12.3%), and Dodecene (9.9%). This research also led to the first identification of the glycoside compound 8-di-C-glucoside, Apigenin-6, in the ethyl acetate extract of the *Astragalus* genus. Furthermore, m-tolualdehyde, acetophenone, and croweacin were found to be the primary components of the essential oil in this species.

It has been examined^101^ the essential oil compositions and antioxidant activities of *Astragalus adscendens* and *Astragalus verus*. Their analysis revealed 25 and 22 compounds in each species, respectively. The dominant compounds were phthalate (59.88%) in *Astragalus adscendens* and phytol (38.02%) in *Astragalus verus*. *Astragalus adscendens* had the highest total flavonoid content at 57.5 mg QE/g DW. It has been investigated^102^ the essential oil compounds in the leaves, roots, and gum of *Astragalus compactus* Lam. They identified a total of 19 compounds across different parts of the plant. The composition and quantity of active ingredients varied significantly between these parts. Notably, only one volatile organic chlorinated compound was detected in the root, and no toxic chlorinated compounds were found in the gum, indicating its high quality. It has been analyzed^103^ the chemical compounds in the fruit of *Astragalus alopecurus* Pall. and identified 44 compounds, which accounted for 94.13% of the total essential oil. The most significant components were α-pinene (18.41%), humulene epoxide II (12.84%), and α-humulene (11.81%). In terms of phytochemical analysis, the concentrations of phenolic, flavonoid, and alkaloid compounds were measured at 53.61, 115.64, and 0.11 mg/g, respectively. Five compounds identified in this study—Tridecane, Tetradecane, Pentadecane, and Heptadecane—were similar to those extracted in our research.

Silver nanoparticles (AgNPs) synthesized from Anzaroot exhibit cytotoxic effects on MCF-7 human breast cancer cells through various mechanisms. The synthesis process begins with an aqueous extract of the plant, which contains phytochemicals that reduce silver nitrate (AgNO_3_) to form AgNPs, resulting in a noticeable color change. Characterization techniques, such as UV–Vis spectroscopy and transmission electron microscopy (TEM), confirm that the nanoparticles typically range in size from 12 to 34 nm. The observed cytotoxicity is associated with oxidative stress caused by reactive oxygen species (ROS), disruption of DNA replication, and damage to cellular membranes. Furthermore, the phytochemicals in the extract not only facilitate the synthesis of the nanoparticles but also enhance their anticancer activity. The cytotoxicity of AgNPs is dose-dependent, suggesting that optimizing concentrations could improve therapeutic efficacy^2^.

Medicinally active plants are the best reservoirs of diverse phytochemicals for green synthesis of AgNPs^104^. AgNPs have shown extraordinary medical potentials as viable anti-tumor drug-delivery frameworks. As already specified, routine cancer therapies such as chemotherapy, radiotherapy or surgery have their restrictions related with cytotoxic effects, uncommon side effects, serious resistance issues and need of specificity. AgNPs overcome these impediments by diminishing the side effects and improving the efficacy of cancer treatment^105^. In general, there are some advantages and disadvantages for green synthesis of nanoparticles. The biologic strategies are reasonable, eco-friendly and non-toxic. While, no complex setup is required, they offer better tuned control of the measure and shape of the NPs compared to chemical and physical strategies. It is not necessary to use stabilizing operators in order to anticipate the aggregation of the NPs. Nevertheless, there are some disadvantages, as well. Synthesis by biologic strategies is not as quick as nucleation by chemical methods. Due to the presence of various biomolecules within biologic sources, it is hard to specify the precise biomolecules involved in the nucleation of NPs. As, organism poisons may be brought together by biologic union, in the event that the biologic union is conducted on a huge scale, some environmental issues may occur due to the abuse of di-verse biologic species.

## Conclusion

The study successfully synthesized silver nanoparticles (AgNPs) using extracts from *Anzaroot*, optimizing conditions such as pH, extract volume, silver nitrate concentration, and reaction time. The ideal parameters for SNP production were determined to be pH 8, 4 ml of extract, and a reaction time of 300 min. Characterization through UV–Vis spectrophotometry, FTIR, and XRD confirmed the successful synthesis and crystalline structure of the nanoparticles. The cytotoxicity assessment on MCF-7 cancer cells revealed significant dose-dependent anticancer activity, particularly with the 5mM AgNO_3_ combined with root extract. The synthesized AgNPs ranged from 12 to 24 nm in size, with phytochemicals enhancing their synthesis rates and biological effects. The IC50 value for root extract-mediated SNPs was found to be 21.73 μg/mL. This research highlights the potential of plant-based methods for synthesizing nanoparticles, suggesting applications in food, pharmaceuticals, and agriculture as effective natural antioxidants. Overall, it emphasizes the promising role of green synthesis in biomedical applications.

## Supplementary Information

Supplementary Information 1.

Supplementary Information 2.

## Data Availability

All the data are embedded in the manuscript.
