# Supplementary material for: Green synthesis of silver nanoparticles from plant Astragalus fasciculifolius Bioss and evaluating cytotoxic effects on MCF7 human breast cancer cells
Source: Sci Rep. 2025 Jul 15;15:25474. doi: 10.1038/s41598-025-05224-5 (PMC12264097; doi:10.1038/s41598-025-05224-5)
Supplement: Supplementary file 1 — Supplementary Information 1. [file 41598_2025_5224_MOESM1_ESM.docx]

| **Table S1. Optimization of the factors affecting SNPs** | | | | |
| --- | --- | --- | --- | --- |
| Investigated factor | Plant extract (ml) | AgNO_3_ volume (ml) | AgNO_3_ (mM) | pH |
| pH | 4 | 10 | 5 | 2 |
|  | 4 | 10 | 5 | 4 |
|  | 4 | 10 | 5 | 6 |
|  | 4 | 10 | 5 | 8 |
|  | 4 | 10 | 10 | 2 |
|  | 4 | 10 | 10 | 4 |
|  | 4 | 10 | 10 | 6 |
|  | 4 | 10 | 10 | 8 |
| Extract volume | 1 | 10 | 5 | 8 |
|  | 2 | 10 | 5 | 8 |
|  | 3 | 10 | 5 | 8 |
|  | 4 | 10 | 5 | 8 |
|  | 1 | 10 | 10 | 8 |
|  | 2 | 10 | 10 | 8 |
|  | 3 | 10 | 10 | 8 |
|  | 4 | 10 | 10 | 8 |
| AgNO_3_ (Mm) | 4 | 10 | 1 | 8 |
|  | 4 | 10 | 5 | 8 |
|  | 4 | 10 | 10 | 8 |
| Reaction time (min) |  |  |  |  |
| 30 | 4 | 10 | 5 | 8 |
| 60 | 4 | 10 | 5 | 8 |
| 300 | 4 | 10 | 5 | 8 |
| 30 | 4 | 10 | 10 | 8 |
| 60 | 4 | 10 | 10 | 8 |
| 300 | 4 | 10 | 10 | 8 |
| * The final volume of SNPs biosynthesis reaction was kept to 20 ml, with water. | | | | |
| *The investigated biosynthesis treatments were as follows: Root extract (**R**), Gum extract (**G**), 1Mm AgNO3+ Root extract (R1); 5Mm AgNO3+ Root extract (**R2**); 1Mm AgNO3+ Gum extract (**G1**), and 5 Mm AgNO3+ Gum extract (**G2**). | | | | |

| **Table S2.** Preparation of solutions of different concentrations was done based on ‘Serial Dilution Method’. | | | |
| --- | --- | --- | --- |
| Medium (mL) | Nanoparticle (Stock, 𝝻g) | | Final volume (𝝻g nanoparticle/mL medium) |
| 4000 | None Stock (full medium) | 4000 | 1000 𝝻g/mL |
| 1000 | First stock | 1000 | 500 𝝻g/mL |
| 3040 | Second Stock | 960 | 120 𝝻g/mL |
| 400 | Third Stock | 2000 | 100 𝝻g/mL |
| 400 | Fourth | 1600 | 80 𝝻g/mL |
| 400 | the fifth | 1200 | 60 𝝻g/mL |
| 400 | the sixth | 800 | 40 𝝻g/mL |
| 600 | the seventh | 600 | 20 𝝻g/mL |
| 400 | Eighth | 800 | 15 𝝻g/mL |
| 250 | ninth | 500 | 10 𝝻g/mL |
| 250 | the tenth | 250 | 5 𝝻g/mL |
| 400 | Control | 0 | 0 𝝻g/mL |
