# Supplementary figures and images for: Green synthesis of silver nanoparticles from plant Astragalus fasciculifolius Bioss and evaluating cytotoxic effects on MCF7 human breast cancer cells
Source: Sci Rep. 2025 Jul 15;15:25474. doi: 10.1038/s41598-025-05224-5 (PMC12264097; doi:10.1038/s41598-025-05224-5)

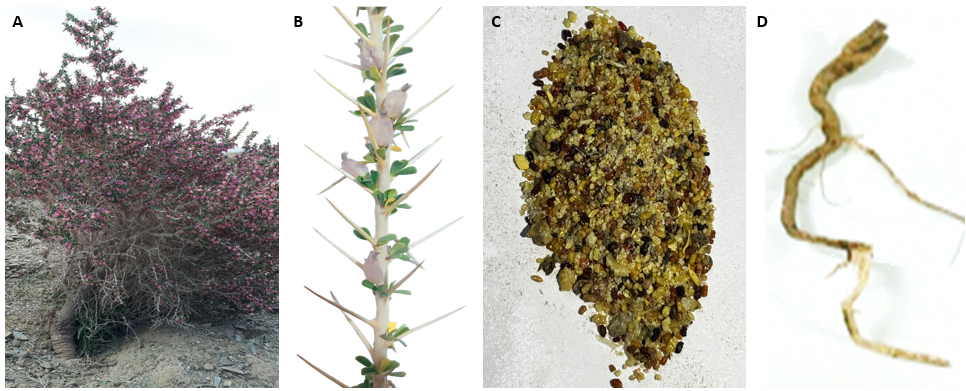


**Figure S1. (A)** The shrub, (**b**), the Stem, leaf and Flower (B), the gum (C) and the Root (D) of *A. fasciculifolius* Bioss.

Supplement: Supplementary file 2 — Supplementary Information 2. [file 41598_2025_5224_MOESM2_ESM.docx]
